# The Report of the Departmental Committee on Poor-Law Orders

**Published:** 1913-08-23

**Authors:** 


### The Hospital

The Workers' Newspaper of Administrative Medicine and Institutional Life, Administration, National Insurance and Health.

No. 1417, Vol. LIV. SATURDAY, AUGUST 23, 1913. PRICE ONE PENNY

### THE REPORT OF THE DEPARTMENTAL COMMITTEE ON POOR-LAW ORDERS.

In January 1911 Mr. John Burns appointed Sir Samuel Butler Provis, the former permanent secretary of the Local Government Board, Sir James Stewart Davy, the chief general inspector, Mr. Jerred, one of the assistant secretaries, and Mr. Thomas Smith, the clerk to the West Ham Guardians, to examine the various Poor-Law Orders, with a view to their consolidation with amendments into one or more new Orders. The first Report of this Committee, embodying proposals for a Poor-Law institutions Order applicable to all Poor-Law institutions, except separate infirmaries, separate schools, and casual wards, has just been published. In dealing with the Report from a medical point of view this limitation in its applicability must always be borne in mind. Its weakness and its strength can be directly traced to the composition of the Committee which framed it. Its strength, as might be expected, lies in the brilliant and statesmanlike manner in which it codifies, simplifies, and improves the numerous Orders relating to the general administration of the mixed workhouse. Its weakness, due to the absence of any member experienced in the medical administration of an institution and with special knowledge of the medical requirements of the sick wards of workhouses, lies in a failure to appreciate the necessity of a drastic reform in the administration of these wards if they are to be brought up to the standard attained by the modern separate Poor-Law infirmary.

Nevertheless, the proposed alterations in the regulations governing their administration are, on the whole, a notable advance upon the present system, and even upon the proposals contained in the draft of the institutions Order circulated privately last year, on which we commented at the time.

As regards the medical officer, his work is to be very seriously increased in many Unions, although in some of the more advanced ones the work which is now to be compulsory upon all is already being carried out. He is to keep a record of the physical and mental condition and fitness for employment of all persons admitted to the workhouse; he is to supply the Guardians with written recommendations before they deal with the classification of certain inmates; he is to keep records of the medical history, diet, and treatment of each inmate of a sick ward or mental ward, and of each infant under the age of eighteen months; he is to examine every infant under eighteen months not less often than once in every two weeks, and every infant above eighteen months and every child at least every month and is to record the result of each examination; he is to furnish the medical officer of health with such information as the Local Government Board may direct with respect to cases of sickness or death within the institution; and he is to supply to the clerk of the Guardians, so far as matters affecting his office are concerned, any information necessary for the purpose of any report, answer, or return required by the Local Government Board or their officers, or by the Guardians or otherwise, required for the business of the Union. The only off-set against all this additional work is that the Medical Relief Register is to be abolished. It is obvious that in large Unions it will be necessary to provide for the purely clerical work to be carried out in the master's office and for an additional assistant medical officer to be appointed, whilst in the smaller Unions it will be necessary to increase the salary of the medical officer to compensate him for the additional time he will have to devote to his duties. But upon all these obvious corollaries of the proposals the Order is silent. The proposed repeated examinations of healthy children are totally unnecessary. What is required is that the children's wards should be under the control of the medical officer; that the children should be under the direct supervision of trained nurses directly responsible to the medical officer; that they should be periodically weighed and measured; and that they should be examined by the medical officer as often as he thinks necessary.

It is to be regretted that there is nothing in the proposed Order to improve the status of the workhouse medical officer. The master is "to govern and control, subject to the directions of the Guardians, the institution and the officers." This wording is more precise than in the old Orders, and to this extent it places the medical officer (and the same remark applies to the superintendent nurse) in a worse position as regards subordination to the master than is the case now. The same vicious system which has given rise to so much friction in the past and which has prevented most well-trained medical men and nurses from applying for these posts is not merely to be perpetuated, but is to be rendered more obnoxious. Moreover, the master is still required, as in the old Orders, to take care that all sick and insane inmates and infants are duly visited by the medical officer. No objection could be taken to a provision that the master should report to the house committee any occasion on which the medical officer failed to attend when properly summoned; but to give these powers of general supervision of a highly-specialised official to an officer in no way qualified to exercise them is to degrade the position of the medical officer. The fact is that the Departmental Committee have undertaken an impossible task. They wish to introduce into the sick wards of the workhouse the same standard of efficiency as has been reached in the separate infirmaries whilst retaining the general administrative control of the workhouse master over all departments of the workhouse. The two objects are incompatible. Drastic changes are required, and the Departmental Committee has not had the courage or has not had the special knowledge necessary to suggest them.

There is another matter of great medical interest in these proposals, and one which runs directly counter to the traditions of the medical profession. It is suggested that it shall be the duty of all officers to produce all books, documents, or accounts kept by them, or in their custody, whenever required by the Guardians, the house committee, or any other duly appointed committee of the Guardians, or the clerk to the Guardians. As a general rule no objection could be taken to this suggestion, but every medical officer has in his possession documents containing statements divulged to him by his patients under the usual conditions governing the relations of patients and their medical men. To produce these documents at the request of the clerk acting without any other authority would be a serious breach of professional etiquette. No conscientious medical officer would feel justified in doing so.

